# Pulmonary Function Testing in Cement Transport Workers at Incheh Borun Border, Northeast of Iran

**Published:** 2019-07

**Authors:** Javad KHADEMI, Mahdi SADEGHI, Rahmatollah AHMADPOOR, Jalalodin TAMADON YOLME, Mohammad Ali MIRZAIE, Nazanin IZADI, Zahra MEHRBAKHSH

**Affiliations:** 1. Department of Public Health, Faculty of Health, Golestan University of Medical Sciences, Gorgan, Iran; 2. Environmental Health Research Center, Golestan University of Medical Sciences, Gorgan, Iran; 3. Department of Environmental Health Engineering, Faculty of Health, Golestan University of Medical Sciences, Gorgan, Iran; 4. Department of Occupational Health Engineering, Health Center of Gorgan, Golestan University of Medical Sciences, Gorgan, Iran; 5. Center for Research on Occupational Diseases, Tehran University of Medical Sciences, Tehran, Iran; 6. Department of Biostatistics, Faculty of Health, Golestan University of Medical Sciences, Gorgan, Iran

**Keywords:** Cement plant, Health effects, Respiratory problems, Pulmonary function tests, Transport workers

## Abstract

**Background:**

The aim of this study was to evaluate respiratory problems via pulmonary function testing in cement transport workers at Incheh Borun border in northeast of Iran in 2016.

**Methods:**

The study was conducted on 358 male workers. All subjects were evaluated for respiratory symptoms via pulmonary function testing and completion of the American Thoracic Society questionnaire.

**Results:**

Mean age of workers was 34.8±12.87 yr (age range 16–79 yr). Mean duration of employment was 6.1±4.36 yr. Most workers (75.4%) were illiterate or had elementary education. In addition, 56 (15.6%) subjects were smokers. Only three individuals (0.3%) had obstructive pattern. There was a significant relationship between age of workers and frequency of respiratory problems (*P*<0.05).

**Conclusion:**

It is necessary to educate the workers about the health and safety regulations and use of personal protective equipment in workplace. In addition, periodic evaluation of respiratory function could help protect workers from developing occupational diseases.

## Introduction

Occupational disease is caused by constant exposure of workers to harmful occupational physical, chemical, biological, ergonomic and psychological factors (1).

Production and transportation of cement could act as important sources of air pollution (2). Transportation of cement by workers has some adverse health effect on the workers. Portland cement has been commonly used worldwide despite its adverse health effects on the workers. Workers in the cement industry including cement carriers and transporter are exposed to cement dust (3).

Exposure of inhaling particulate materials that may lead to adverse respiratory effects (3,4). Skin disease, lung function deterioration, sputum, coughing, wheezing and increased incidence of chronic obstructive pulmonary diseases have been reported to be associated with exposure to cement dust (3, 5–7). In addition to smoking, several other risk factors are involved in development of lung cancer as well as another type of cancer (3, 4, 8).

Occupational exposure to pollutants is considered as a risk factor in 13%–29% of patients. Several important occupational carcinogenic agents including arsenic, asbestos, beryllium, cadmium, chromium, nickel, silica and particulate matter (in high concentrations) and volatile organic compounds (VOCs) have been identified so far (9,10).

Community based studies have showed increased relative risks for respiratory symptoms consistent with chronic obstructive pulmonary disease (COPD) as well as for excess annual declines in Forced Expiratory Volume in one second (FEV1) associated with occupational exposure to dust or gases and other pollutants (4).

Among harmful agents in workplace, those that cause respiratory tract problems are the most common cause of occupational diseases. In occupational respiratory diseases, spirometric pulmonary function tests can detect lung dysfunction before the onset of clinical symptoms and describes the effect of restriction or obstruction on the lung function (11,12). It is also used for screening workers with exposures to agents and pollutants associated with pulmonary diseases. Lung function is influenced by factors such as sex, age, weight, smoking, duration of employment, environment and ethnicity (4, 13). Lung function test provides a clearer understanding of pulmonary function in subjects of different occupation and profession (4). The American Thoracic Society (ATS) recommends that procedural source of variation in lung function be minimized (11).

Several studies have evaluated the effects of cement dust exposure on development of lung diseases (8, 14,15). In a similar study on respiratory symptoms and pulmonary function tests observed among construction and sanitary workers of Thoothukudi. Overall, 249 workers turned up. After considering the exclusion criteria, 101 construction workers and 56 sanitary workers in the study group were compared with 92 controls in terms of their respiratory status. Respiratory complaints were significantly higher in the study group compared to controls. Frequencies of abnormal spirometric findings were significantly higher in the study group. Occupational exposure to cement, road dust and unwanted wastes created severe harm to the worker’s respiratory system (4).

Another research conducted on dust exposure and respiratory health effect in cement production. A respiratory symptoms questionnaire was completed and pulmonary function tests were carried out on 94 exposed and 54 non exposed workers at a cement factory in the east of Iran. In this study cough, sputum, wheezing and dyspnea were more prevalent among exposed subjects. Exposed workers compared to the unexposed group showed significant reduction in Forced Expiratory Volume in one second (FEV1), Forced Vital Capacity (FVC), and Forced Expiratory Flow between 25% and 75% of the FVC (FEF25–75%) (P<0.05). There was direct association between cement dust exposure and functional impairment among the cement factory workers (16).

Iran-Turkmenistan border located in the northeast of Iran is a target point for transit of various goods and materials such as cement to neighboring countries. Workers are usually responsible for moving around cement and similar materials at the border. Moreover, they have not undergone medical screening or testing. Therefore, this study aimed to evaluate the respiratory and pulmonary function of cement transport workers at Incheh Borun border in the northeast of Iran.

## Materials and Methods

This descriptive-analytical cross-sectional study was conducted in 2016, on 358 male cement transport workers at the Incheh Borun border, located in the northeast of Iran. All subjects were evaluated for respiratory symptoms via pulmonary function testing. Data were collected by ATS standard questionnaire (17,18) and physical examination. Spirometric parameters including forced vital capacity (FVC), forced expiratory volume in one second (FEV1), FEV1/FVC ratio and peak expiratory flow rate (PEFR) were also measured.

The study was approved by the Ethics Committee of the Golestan University of Medical Sciences (IRCT code: IR.goums.REC.1394.20). Written informed consent was obtained from all participants before the study.

The questionnaire included questions regarding demographic information, employment history, smoking habits and respiratory problems such as cough, sputum, dyspnea and chronic bronchitis (productive cough three months per year for two consecutive years), and throat, nose and eye irritation or burning.Pulmonary function tests were carried out by a trained technician using a calibrated spirometry apparatus (Spirolab 2), between 8 and 12 a.m. before the workers start work. At least three respiratory maneuvers were taken, and the best results were evaluated according to the ATS criteria (18).

### Statistical analysis

Data were analyzed by SPSS (ver. 18, Chicago, IL, USA). Quantitative and qualitative variables were measured. Chi-square and Mann Whitney tests were used to determining the association between two qualitative variables. Independent sample t-test was used to evaluate differences between the quantitative variables in exposed and non-exposed subjects. *P*-values less than 0.05 were considered statistically significant.

## Results

Demographic and occupational characteristics of the subjects were presented in Table 1.

**Table 1:** Demographic and occupational characteristics of the subjects

***Characteristics***	***Mean***	***Stamdard Deviation (SD)***	***Minimum***	***Maximum***
Age (year)	34.8	12.87	16	79
Height (cm)	175	6.6	150	205
Weight (Kg)	74.6	12.48	44	120
Duration of employment (year)	6.1	4.36	1	18
BMI	24.3	3.8	16.14	43.34

Mean of age was 34.8 ± 12.87 yr (age range 16–79 yr) working at the Incheh Borun border, Iran. Information and work habits of cement transporters working at the Incheh Borun border were presented in Table 2.

**Table 2:** Information and work habits of cement transporters working at the Incheh Borun border

***Variable***	***Status***	***Number***	***Percent***
Marital status	Married	276	77.1
	Single	82	22.9
Level of Education	Illiterate	125	34.9
	Primary	145	40.5
	Diploma and higher	80	22.3
	Graduate	8	2.2
Use of personal protective equipment	Yes	23	6.4
	No	335	93.6
Smoking	Yes	56	15.6
	No	302	84.4
The number of cigarettes smoked per day	0	302	84.4
	<3	29	8.1
	≥3	27	7.5

The mean duration of employment was 6.1±4.36 yr. Most workers (75.4%) were either illiterate or had elementary education. In addition, 302 (84.4%) subjects were non-smoker and 56 (15.6%) were smoker.

The result of pulmonary function testing is shown in Table 3. Of 358 subjects, three individuals (0.3%) had obstructive pattern on spirometry. Symptoms such as sputum, cough, wheezing and shortness of breath were found in 25%, 17%, 14.5% and 6.7% of workers, respectively.

The relationship between mean of age and respiratory problems were presented in Table 4. There was a statistically significant relationship between age and frequency of respiratory problems.

**Table 3:** Results of pulmonary function test in subjects

***Parameter***	***Mean***	***SD***	***Minimum***	***Maximum***
FVC	83.0894	17.99993	153.00	27.00
FEV1	91.2067	17.43870	148.00	32.00
PEF	77.7374	16.93300	135.00	24.00
FEV1/FVC ratio	110.79	91.37	134	69

**Table 4:** The relationship between mean of age and respiratory problems

***Respiratory problems***	***Mean ± SD***	P-value*
Shortness of Breath	40.58±2.73	0.015
Shortness of breath while walking on a flat surface	41.31±2.94	0.012
Shortness of breath after stopping	41.8±3.04	0.009
Feeling of tightening in the chest and wheezing	39.19±1.9	0.007
Continuous wheezing	38.58±2.04	0.038
Wheezing during walking	38.47±1.9	0.038
Wheezing during running	38.94±15.09	0.035
Wheezing during sleep	38.46±14.05	0.038
Wheezing throughout the day	38.46±14.05	0.037
Feel Wheezing during walking	8.15±5.43	0.004
Feel Wheezing during running	8.17±5.44	0.004
Feel Wheezing during sleep	8.26±5.44	0.003
Feel Wheezing throughout the day	8.26±5.44	0.002

Mann-Whitney test

Respiratory problems were more prevalent in older workers. The relationship between demographic information and respiratory problems were presented in Table 5. There were statistically significant relationships between the duration of employment, level of education and most respiratory problems.

**Table 5:** The relationship between demographic information and respiratory problems

***Respiratory problems***	***Level of Education***	***Use of personal protective equipment***	***Age(yr)***	***Duration of employment***	***BMI***
Cough	*P*<0.001	*P*=0.59	*P*=0.144	*P*=0.049	*P*=0.19
Cough When waking	*P*<0.029	*P*=0.78	*P*=0.85	*P*=0.048	*P*=0.46
Coughing for more than three consecutive months	*P*<0.001	*P*=0.67	*P*=0.62	*P*=0.011	*P*=0.3
Sputum	*P*<0.004	*P*=0.4	*P*= 0.7	*P*=0.024	*P*=0.002
Sputum during sleep	*P*<0.004	*P*=0.14	*P*=0.17	*P*=0.01	*P*=0.37
Sputum for more than three months	*P*<0.001	*P*=0.57	*P*=0.96	*P*=0.004	*P*=0.013
Sputum amd coughing attack	*P*<0.001	*P*=0.074	*P*=0.3	*P*=0.029	*P*=0.23
Shortness of breath	*P*<0.01	*P*=0.69	*P*=0.015	*P*=0.06	*P*=0.64
Shortness of breath while walking on a flat surface	*P*<0.014	*P*=0.59	*P*=0.012	*P*=0.016	*P*=0.62
Shortness of breath after walking	*P*<0.007	*P*=0.55	*P*=0.009	*P*=0.005	*P*=0.37
Wheezing during the day	*P*<0.001	*P*=0.83	*P*=0.08	*P*=0.018	*P*=0.19
Wheezing during the week	*P*<0.001	*P*=0.89	*P*=0.039	*P*=0.005	*P*=0.21
Wheezing in the chest	*P*<0.001	*P*=0.84	*P*=0.007	*P*=0.008	*P*=0.28
Continuous wheezing	*P*<0.001	*P*=0.95	*P*=0.038	*P*=0.001	*P*=0.2
Wheezing during walking	*P*<0.001	*P*=0.86	*P*=0.038	*P*=0.004	*P*=0.16
Wheezing during running	*P*<0.001	*P*=0.86	*P*=0.035	*P*=0.004	*P*=0.17
Wheezing during sleep	*P*<0.001	*P*=0.92	*P*=0.038	*P*=0.003	*P*=0.2
Wheezing throughout the day	*P*<0.001	*P*=0.92	*P*=0.037	*P*=0.002	*P*=0.2
Smoking	*P*<0.034	*P*=0.34	*P*=0.6	*P*=0.076	*P*=0.85

The relationship between variables and pulmonary function test parameters were presented in Table 6. There was a statistically significant relationship between pulmonary function test variables (except for FEV1) and the level of education.

**Table 6:** The relationship between variables and pulmonary function test parameters

***Spirometric parameters***	***Use of personal protective equipment***	***marital status***	***Level of Education***	***BMI***
FVC	*P*= 0.042	*P*= 0.011	*P*= 0.05	*P*= 0.96
FEV1	*P*= 0.08	*P*= 0.08	*P*= 0.36	*P*= 0.84
PEF	*P*= 0.97	*P*= 0.001	*P*= 0.001	*P*= 0.21
FEV1/FVC ratio	*P*= 0.006	*P*= 0.002	*P*= 0.001	*P*= 0.98

## Discussion

In this study, we evaluated the prevalence of respiratory problems in workers at the Incheh Borun border. There was a significant positive correlation between age and respiratory problems such as shortness of breath and wheezing. Moreover, older age was associated with higher frequency of respiratory problems. These results are in agreement with another result (19). Age was reported as an important lung function predictor both before and after reversibility testing and had a significant effect on bronchodilator response of FEV1 and FEV1/FVC ratio (19).

The result of Mann-Whitney test showed a significant relationship between duration of employment and incidence of respiratory problems such as shortness of breath, cough, sputum and wheezing. The duration of employment in the subjects of our study was between 1 and 18 yr. These results are consistent with another study of (20).

In this study, the prevalence of obstructive respiratory disease was significantly (*P*=0.034) higher in the exposed group (20). In another research was conducted among transit and non-transit workers in Nigeria. In this study significant factor associated with higher odds for pulmonary function impairment among transit workers was the duration on job. Transit workers with more than 5 yr on the job had higher odds for incident pulmonary function impairment. This could probably be due to the accumulated effect of air pollutants and other confounding factors (21).

The results also indicated a significant correlation between level of education and frequency of respiratory problems including shortness of breath, cough, and sputum and wheezing. Most workers (75.4%) were either illiterate or had elementary education. Thus, the majority of workers did not have adequate knowledge regarding occupational health and safety guidelines.

Furthermore, there was a significant relationship between sputum, sputum for more than three months and BMI of workers in a way that subjects with higher BMI values had higher volume of sputum (*P*<0.05). Our results were in agreement with the data obtained for relationship between asthma severity and obesity that the results showed a linear relationship between asthma severity and BMI. The prevalence of obesity in the 13 patients on long-term oral corticosteroids was 100%. Prevalence of obesity increases with increasing asthma severity in adults (22).

Furthermore, there was a significant relationship between usage of personal protective equipment (PPE), FVC and FEV1/FVC ratio (*P*<0.05). Our results were in agreement with the data obtained for occupational exposure to respirable suspended particulate matter and lung functions deterioration of steel workers: an exploratory study in India. The majority of the workers were not wearing proper PPEs like masks due to ergonomically bad design. Therefore, the workers were under higher risk of lungs functions deterioration (23).

Occupational and environmental exposure to dust particles and their effects on health is an important problem, especially in developing countries because of the lack of medical education and facilities. In addition, the systematic record-keeping and the difficulty in obtaining measurements at worksites are major hindrances that have made epidemiological research difficult in these countries (24).

Based on the findings of our study, it is necessary to educate workers in similar work environments to change their lifestyle and comply with the occupational health and safety guidelines.

## Conclusion

The cement transport workers at the Incheh Borun border are not under any type of insurance. Most of the workers are illiterate or have elementary education. Therefore, it is necessary to educate workers in similar work environments to change their lifestyle and comply with occupational health and safety guidelines. Periodic checkups and health screenings are recommended to prevent development of occupational diseases in workers at risk.

## Ethical considerations

Ethical issues (Including plagiarism, informed consent, misconduct, data fabrication and/or falsification, double publication and/or submission, redundancy, etc.) have been completely observed by the authors.

## References

[1] Nelson DI Concha-Barrientos M Driscoll T (2005). The global burden of selected occupational diseases and injury risks: Methodology and summary. Am J Ind Med, 48(6):400–18. 16299700 10.1002/ajim.20211

[2] Bertoldi M Borgini A Tittarelli A (2012). Health effects for the population living near a cement plant: An epidemiological assessment. Environ Int, 41:1–7. 22245540 10.1016/j.envint.2011.12.005

[3] Koh DH Kim TW Jang SH Ryu HW (2011). Cancer Mortality and Incidence in Cement Industry Workers in Korea. Saf Health Work, 2(3):243–9. 22953208 10.5491/SHAW.2011.2.3.243 PMC3430901

[4] Mariammal T Amutha A Sornaraj R (2012). Work related respiratory symptoms and pulmonary function tests observed among construction and sanitary workers of Thoothukudi. Int J Pharm tech Res, 4(3):1266–73.

[5] Mwaiselage J Bråtveit M Moen BE Mashalla Y (2005). Respiratory symptoms and chronic obstructive pulmonary disease among cement factory workers. Scand J Work Environ Health, 31(4):316–23. 16161715 10.5271/sjweh.888

[6] Zeleke ZK Moen BE Bråtveit M (2010). Cement dust exposure and acute lung function: a cross shift study. BMC Pulm Med, 10:19. 20398255 10.1186/1471-2466-10-19 PMC2865447

[7] Ahmed HO Abdullah AA (2012). Dust exposure and respiratory symptoms among cement factory workers in the United Arab Emirates. Ind Health, 50(3):214–22. 22453209 10.2486/indhealth.ms1320

[8] Jakobsson K Rannug A Alexandrie AK (1995). Radiographic changes and lung function in relation to activity of the glutathione transferases theta and mu among asbestos cement workers. Toxicol Lett, 77(1–3):363–9. 7618163 10.1016/0378-4274(95)03319-x

[9] Rajer M Zwitter M Rajer B (2014). Pollution in the working place and social status: Co-factors in lung cancer carcinogenesis. Lung Cancer, 85(3):346–50. 24999084 10.1016/j.lungcan.2014.06.012

[10] Kargar M Nadafi K Nabizadeh R (2013). Survey of hazardous organic compounds in the groundwater, air and wastewater effluents near the Tehran automobile industry. Bull Environ Contam Toxicol, 90(2):155–9. 23160750 10.1007/s00128-012-0890-6

[11] Enright PL Beck KC Sherrill DL (2004). Repeatability of spirometry in 18,000 adult patients. Am J Respir Crit Care Med, 169(2):235–8. 14604836 10.1164/rccm.200204-347OC

[12] Wagner N Beckett W Steinberg R (2006). Using spirometry results in occupational medicine and research: Common errors and good practice in statistical analysis and reporting. Indian J Occup Environ Med, 10:5–10.

[13] Mashalla YJ Masesa PC (1992). Changing relationship between FEV1 and height during adolescence. East Afr Med J, 69(5):240–3. 1644040

[14] Smailyte G Kurtinaitis J Andersen A (2004). Mortality and cancer incidence among Lithuanian cement producing workers. Occup Environ Med, 61(6):529–34. 15150393 10.1136/oem.2003.009936 PMC1763656

[15] Vestbo J Knudsen KM Raffn E (1991). Exposure to cement dust at a Portland cement factory and the risk of cancer. Br J Ind Med, 48(12):803–7. 1772795 10.1136/oem.48.12.803 PMC1035459

[16] Kakooe HAG Ghasemkhani AA Hossaini MA Mostafa (2012). Survey of Exposure to Cement Dust and Its Effect on Respiratory Function in Workers of a Cement Complex. Horizon Med Sci, 18(1): 60–5.

[17] Ferris BG (1978). Epidemiology Standardization Project (American Thoracic Society). Am Rev Respir Dis, 118(6 Pt 2):1–120. 742764

[18] Pellegrino R Viegi G Brusasco V (2005). Interpretative strategies for lung function tests. Eur Respir J, 26(5):948–68. 16264058 10.1183/09031936.05.00035205

[19] Johannessen A Lehmann S Omenaas ER (2006). Post-bronchodilator spirometry reference values in adults and implications for disease management. Am J Respir Crit Care Med, 173(12):1316–25. 16556696 10.1164/rccm.200601-023OC

[20] Aminian O Zeinodin H Sadeghniiat-Haghighi K Izadi N (2015). Respiratory Symptoms and Pulmonary Function Tests among Galvanized Workers Exposed To Zinc Oxide. J Res Health Sci, 15(3):159–62. 26411661

[21] Ekpenyong CE Ettebong EO Akpan EE (2012). Urban city transportation mode and respiratory health effect of air pollution: a cross-sectional study among transit and non-transit workers in Nigeria. BMJ Open, 2(5):e001253. 10.1136/bmjopen-2012-001253 PMC3488752 23065446

[22] Akerman MJ Calacanis CM Madsen MK (2004). Relationship between asthma severity and obesity. J Asthma, 41(5):521–6. 15360059 10.1081/jas-120037651

[23] Singh LP Bhardwaj A Deepak KK (2013). Occupational exposure to respirable suspended particulate matter and lung functions deterioration of steel workers: An exploratory study in India. ISRN Public Health, 2013:325410.

[24] Meo SA Azeem MA Ghori MG Subhan MM (2002). Lung function and surface electromyography of intercostal muscles in cement mill workers. Int J Occup Med Environ Health,15(3):279–87. 12462455

